# The integrity of cochlear hair cells is established and maintained through the localization of Dia1 at apical junctional complexes and stereocilia

**DOI:** 10.1038/s41419-020-02743-z

**Published:** 2020-07-16

**Authors:** Yuzuru Ninoyu, Hirofumi Sakaguchi, Chen Lin, Toshiaki Suzuki, Shigeru Hirano, Yasuo Hisa, Naoaki Saito, Takehiko Ueyama

**Affiliations:** 1 Laboratory of Molecular Pharmacology, Biosignal Research Center, Kobe University, Kobe, 657-8501 Japan; 2 Department of Otolaryngology-Head and Neck Surgery, Kyoto Prefectural University of Medicine, Kyoto, 602-8566 Japan

**Keywords:** Hair cell, Paediatric neurological disorders

## Abstract

Dia1, which belongs to the diaphanous-related formin family, influences a variety of cellular processes through straight actin elongation activity. Recently, novel DIA1 mutants such as p.R1213X (p.R1204X) and p.A265S, have been reported to cause an autosomal dominant sensorineural hearing loss (DFNA1). Additionally, active DIA1 mutants induce progressive hearing loss in a gain-of-function manner. However, the subcellular localization and pathological function of DIA1(R1213X/R1204X) remains unknown. In the present study, we demonstrated the localization of endogenous Dia1 and the constitutively active DIA1 mutant in the cochlea, using transgenic mice expressing FLAG-tagged DIA1(R1204X) (*DIA1*-TG). Endogenous Dia1 and the DIA1 mutant were regionally expressed at the organ of Corti and the spiral ganglion from early life; alongside cochlear maturation, they became localized at the apical junctional complexes (AJCs) between hair cells (HCs) and supporting cells (SCs). To investigate HC vulnerability in the *DIA1*-TG mice, we exposed 4-week-old mice to moderate noise, which induced temporary threshold shifts with cochlear synaptopathy and ultrastructural changes in stereocilia 4 weeks post noise exposure. Furthermore, we established a knock-in (KI) mouse line expressing AcGFP-tagged DIA1(R1213X) (*DIA1*-KI) and confirmed mutant localization at AJCs and the tips of stereocilia in HCs. In MDCK^AcGFP-DIA1(R1213X)^ cells with stable expression of AcGFP-DIA1(R1213X), AcGFP-DIA1(R1213X) revealed marked localization at microvilli on the apical surface of cells and decreased localization at cell-cell junctions. The *DIA1*-TG mice demonstrated hazy and ruffled circumferential actin belts at AJCs and abnormal stereocilia accompanied with HC loss at 5 months of age. In conclusion, Dia1 plays a pivotal role in the development and maintenance of AJCs and stereocilia, ensuring cochlear and HC integrity. Subclinical/latent vulnerability of HCs may be the cause of progressive hearing loss in DFNA1 patients, thus suggesting new therapeutic targets for preventing HC degeneration and progressive hearing loss associated with DFNA1.

## Introduction

The actin cytoskeleton is essential for the structural integrity of living cells, and promotes numerous intracellular signaling pathways^1^. Actin monomers form a polarized filament through assembly and disassembly, which is regulated by numerous actin-binding proteins. Apical junctional complexes (AJCs), which are actin-based structures comprising tight junctions (TJs) and adherens junctions (AJs)^2^, are located at the apical surface of polarized epithelial cells, and contribute to cell polarity, cell adhesion, and paracellular permeability^3^.

Inner hair cells (IHCs) and outer hair cells (OHCs) are specialized sensory cells for detecting hearing in the cochlea. Exquisitely organized actin bundles of microvillus-like stereocilia project from the apical surface of hair cells (HCs), and are crucial for transducing sound vibration into glutamate neurotransmitter release between HCs and the terminal of auditory nerve fibers (mechanoelectrical transduction). HCs and supporting cells (SCs) are incorporated into the roof of the organ of Corti (OC) as a robust epithelial barrier, which is sealed by AJCs to produce the electrochemical gradient essential for the mechanoelectrical transduction^2,4,5^. The synaptic ribbon, a specialized neurotransmitter vesicle for complex sense perception tethered at the presynaptic side of the synapse, enables rapid and sustained signal transmission in HCs^6^. The ribbon synapse primarily comprises C-terminal-binding protein 2 (CtBP2) and RIBEYE (a splice variant of CtBP2)^6–8^. Proteome analysis has shown that actin filaments are directly associated with RIBEYE in the cochlea^9,10^ and the cortical actin meshwork regulates synaptic vesicle release at the presynaptic active zone^11,12^. Since mammalian cochlear HCs do not regenerate after birth, HC preservation is important to prevent progressive sensorineural hearing loss (SNHL). However, no effective intervention for maintaining HCs exists.

The mammalian diaphanous-related formin family encompasses three isoforms, Dia1, Dia2, and Dia3, and promotes nucleation of straight actin filaments at the barbed end^13^. mDia1 and mDia2 are the mouse orthologs of human DIA1 (DIAPH1) and DIA3 (DIAPH3), respectively. mDia1–3 is characterized by intramolecular auto-inhibitory interactions between the N-terminal diaphanous inhibitory domain (DID) and the C-terminal diaphanous autoregulatory domain (DAD). Dia1, a well-known downstream target of RhoA^14,15^, is involved in a variety of cellular processes and functions, including mechanotransduction^16,17^, cell polarization^18^, microtubule stabilization^19,20^, axonogenesis of neurons^21^, and exocrine and vesicle trafficking^22^.

DIA1 and DIA3 are associated with the causative genes of hereditary SNHL, DFNA1 (deafness, non-syndromic autosomal dominant, the first type)^23^, and AUNA1 (auditory neuropathy, non-syndromic autosomal dominant, the first type)^24^, respectively. Recently, novel mutants of DIA1, p.R1213X in DIA1–1 and p.R1204X in DIA1-2^25^, have been reported in European^26,27^ and Japanese deaf families^28,29^ with macrothrombocytopenia. Additionally, p.A1210S*fs*X31^27^, p.A1210G*fs*X31^30^, p.E1192_Q1220del^30^, and p.E1184A*fs*X11^31^ mutants of DIA1 have been reported. Mutations located in or just before the DAD (amino acid [aa] 1197–1217) result in truncation or deletion of the DAD, insufficient auto-inhibition of DIA1, and upregulated actin nucleation without regulation by RhoA^28^. A mutation in the DID of DIA1, p.A265S, located at the pocket formation in the DID accepting the DAD, leads to disruption of the auto-inhibitory DID-DAD interaction, making DIA1 constitutively active^32^. Using a transgenic mouse model of DFNA1 expressing the FLAG-tagged DIA1(R1204X) mutant, *DIA1*^*TG*^, we previously found that basal turn-dominant OHC degeneration causes progressive HL, beginning in the high-frequency range^28^. Although these findings have provided insights into a gain-of-function mechanism of DFNA1, the physiological function of endogenous Dia1 in the cochlea, and in which cells and how the constitutively active mutants create the phenotype, remains unknown.

We hypothesized that Dia1 contributes to actin cytoskeletal integrity in cochlear cells, and that excessive/disorganized actin turnover by DIA1 mutants alters cell shapes, affecting the integrity of highly polarized cochlear cells, including HCs. To prove the hypothesis, we investigated the localization of endogenous Dia1 and the ultrastructural changes in the cochlea using *DIA1*^*TG/TG*^ (*DIA1*-TG) mice. Additionally, we generated another mouse model of DFNA1 expressing an AcGFP-tagged DIA1(R1213X) mutant instead of endogenous Dia1, *DIA1*^*KI/KI*^ (*DIA1*-KI), to investigate precisely where in the cochlea DIA1(R1213X) functions. We found endogenous Dia1 was expressed during and after the differentiation of the OC sensory epithelium and spiral ganglion neurons (SGNs), and was localized at AJCs. The constitutively active mutant accumulated at HC AJCs and stereocilia tips in the *DIA1*-KI mice. Ultrastructural changes in the AJCs and stereocilia were characterized in *DIA1-*TG mice. In addition, *DIA1-*TG mice showed stereocilia deformities and cochlear synaptopathy after noise exposure (NE), suggesting subclinical vulnerability of HCs. Thus, Dia1 strongly influences the development and maintenance of HC structure and integrity, providing new insights into the underlying pathogenic mechanisms of DFNA1.

## Results

### Dia1 expression in the organ of Corti and spiral ganglion of wild type and transgenic *DIA1* mice

**Fig. 1:**
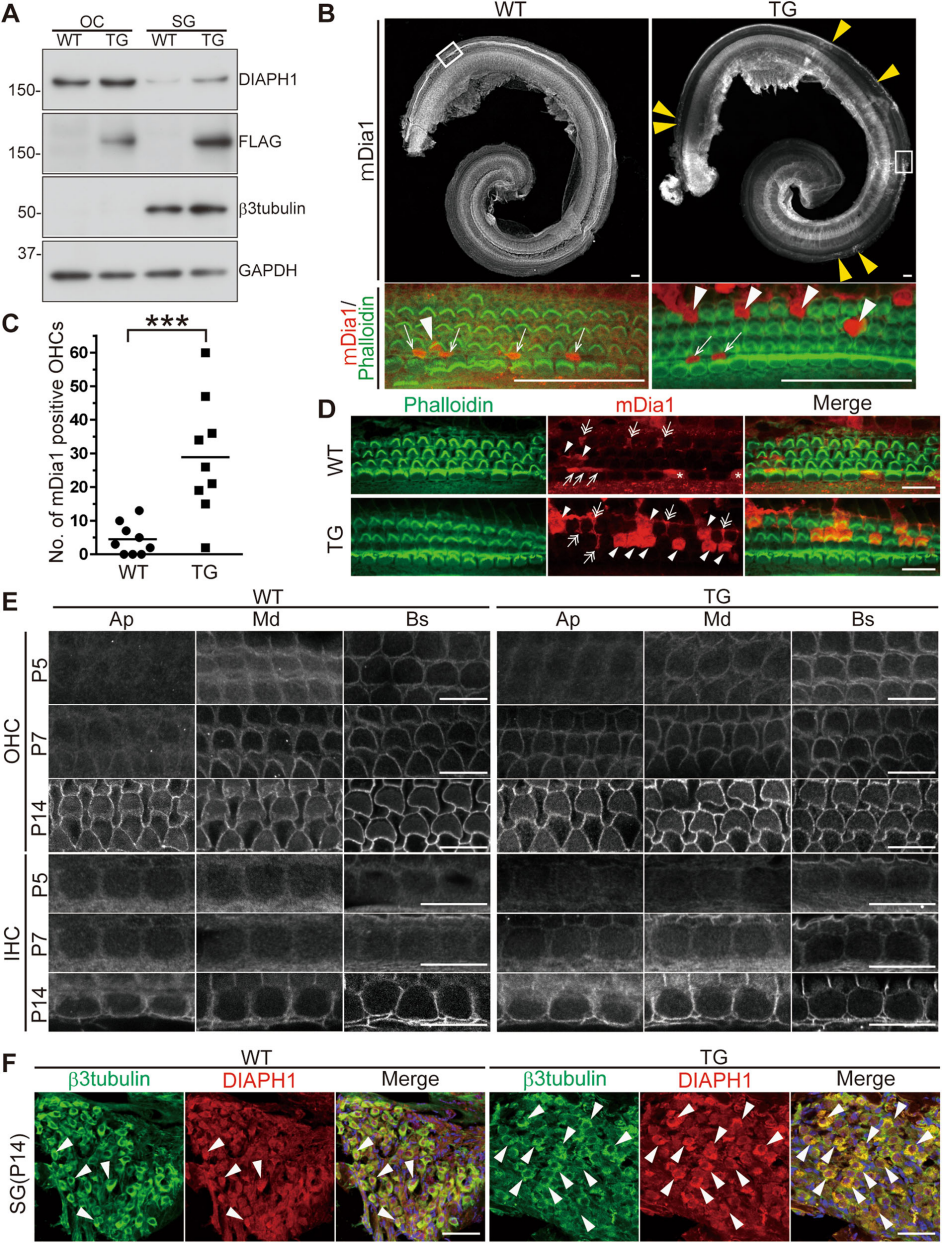
Immunolocalization of Dia1 in the organ of Corti and spiral ganglion. **a** Lysates were obtained from the organ of Corti (OC) and the spiral ganglion (SG) of WT and *DIA1*^*TG/TG*^ (TG) mice at P5. Expression of FLAG-tagged DIA1(R1204X) was confirmed by immunoblotting using FLAG and DIAPH1 antibodies. Comparable loading of proteins was confirmed using β3-tubulin and GAPDH antibodies. Uncropped images are shown in Fig. S1A. **b** Cochleae were obtained from WT and TG mice at P5, and immunostained using an mDia1 antibody followed by an Alexa568-conjugated secondary antibody and Alexa488-conjugated phalloidin. Arrowheads in the low-magnification view of the TG cochlea show the dense region of mDia1-positive cells, which was not detectable in WT mice. High magnification views of the OC from the boxed region in the upper panels are shown in lower panels (mDia1: red, phalloidin: green). Arrows and arrowheads show mDia1-positive inner pillar cells (IPCs) and outer hair cells (OHCs), respectively. Scale bars: 50 μm. Mid-modiolar-section images of the cochlea from TG mice at P8 are shown in Fig. S2A–C. **c** Statistical analysis of the number of mDia1-positive OHCs in the OC of WT and TG mice at P5 (*n* = 9). ***p = 0.0009 by unpaired Student’s *t-*test. **d** High magnification views of the OC from WT and TG mice at P6. Arrowheads, double-headed arrows, arrows, and asterisks show mDia1-positive OHCs, Deiters’ cells (DCs), IPCs, and inner hair cells (IHCs), respectively. Scale bars: 20 μm. Reconstructed lateral projections of the OC from TG mice at P5 are shown in Fig. S2D–F. **e** Whole-mount preparations of the OC dissected into three segments (Ap: apical turn, Md: middle turn, and Bs: basal turn) were obtained from WT and TG mice at P5, P7, and P14, and stained using an mDia1 antibody. Each panel shows a confocal microscopic image focused at an AJC plane containing both OHCs and IHCs. A basal-to-apical progressive increase in the immunoreactivity of mDia1 was observed, as seen with maturation of the OC. Scale bar: 10 μm. **f** Cryostat sections of cochleae at the SG plane from WT and TG mice were obtained at P14, and immunostained using DIAPH1 (red) and β3-tubulin (green) antibodies with DAPI (blue). DIAPH1 immunoreactivity was enhanced using tyramide signal amplification (TSA). Arrowheads indicate DIAPH1-highly positive cells. Scale bar: 50 μm.

To investigate the cochlear expression pattern of Dia1 in wild type (WT) and *DIA1*^*TG/TG*^ transgenic (TG) mice, we performed immunoblotting (IB) and immunofluorescence imaging of cochleae using a DIAPH1 (Abcam, Cambridge, UK) or mDia1 (BD Biosciences, Franklin Lakes, NJ, USA) antibody, whose immunogens are human DIA1 and mouse Dia1, respectively. IB using the DIAPH1 antibody revealed that endogenous Dia1 was expressed in the OC and the spiral ganglion (SG) at postnatal day 5 (P5) (Fig. 1a and Fig. S1). Overexpression of FLAG-DIA1(R1204X) in the OC and SG of TG mice was also confirmed using a FLAG antibody (Fig. 1a and Fig. S1). In the low-magnification view of the P5 mDia1-immunostained cochleae, the immunolabelled cells were loco-regionally observed in TG mice, but were undetectable in WT mice (Fig. 1b). In the high-magnification view of the OC, OHCs and inner pillar cells (IPCs) were stained (Fig. 1b and Fig. S2). The number of immunolabelled OHCs was significantly increased in TG mice compared to WT mice (Fig. 1b–d), suggesting overexpression of FLAG-DIA1(R1204X) in TG mice. The expression of Dia1 was observed not only in OHCs and IPCs, but also in other cell types of the OC, such as Deiters’ cells (DCs) and outer pillar cells (OPCs), in both WT and TG mice (Fig. 1d and Fig. S2). Immunolabelled IHCs were observed in both WT and TG mice, but much more infrequently than the other cell types. Immunolabelled cell types in WT and TG mice were similar (Fig. 1b, d).

Next, we investigated developmental changes in mDia1 immunoreactivity within OHC and IHC AJCs at different ages (P5, P7, and P14; Fig. 1e). mDia1 was first detected in OHCs at the basal turn in both WT and TG mice at P5, and the intensity increased and spread in a basal-to-apical manner, as per OC maturation. The incorporation of mDia1 into AJCs of OHCs preceded its incorporation into IHCs.

Finally, immunostaining of cryostat cochlear sections was performed to examine Dia1 expression in the SGNs of mature cochleae at P14 in WT and TG mice. To detect the weak signal of Dia1, the DIAPH1 antibody was augmented using tyramide signal amplification (TSA) because it was more sensitive than the mDia1 antibody to the amplifying method. This revealed a larger number of DIAPH1-positive SGNs in TG mice compared to WT mice (Fig. 1f); the signal was primarily located in the soma of SGNs.

### Vulnerability to NE in *DIA1*-TG mice

**Fig. 2:**
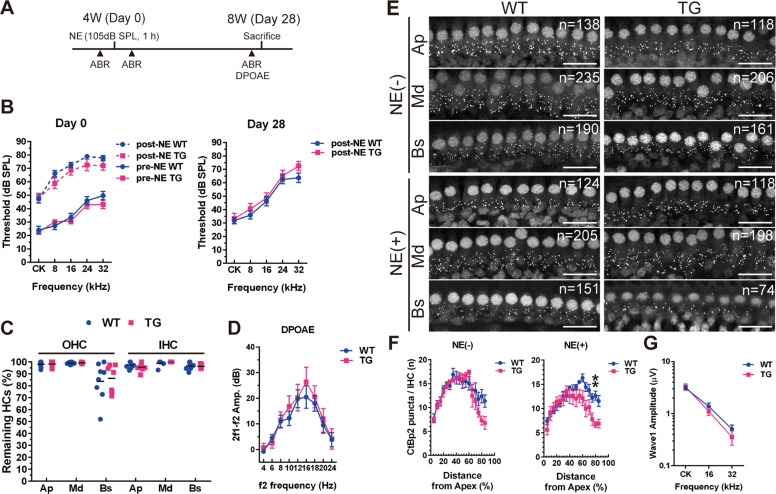
Vulnerability of ribbon synapses to noise exposure in *DIA1*-TG mice. **a** The protocol for the noise exposure (NE) experiments is illustrated. **b** Auditory brainstem response (ABR) thresholds (dB sound pressure level [SPL]) at click (CK), 8, 16, 24, and 32 kHz in WT and *DIA1*^*TG/TG*^ (TG) mice were measured at the age of 4 weeks (4 W, just before and after NE at day 0) and 8 weeks (8 W, post-NE at day 28). Note the NE-induced a temporary threshold shift (TTS) both in WT and TG mice (*n* = 14). **c** The percentages of remaining inner hair cells (IHCs) and outer hair cells (OHCs) at each cochlear turn (Ap: apical, Md: middle, and Bs: basal turn) in WT and TG mice at day 28 after NE. There was no significant difference between WT (*n* = 8) and TG mice (*n* = 7). **d** Distortion product otoacoustic emission (DPOAE) measurement with pure-tone bursts at 4, 6, 8, 10, 12, 16, 18, 20, and 24 kHz at day 28 after NE. There was no significant difference between WT and TG mice (*n* = 8). **e** At day 28 after NE experiments (at 8-weeks-old), three turns of the cochlea (Ap, Md, and Bs) were obtained, and immunostained using a CtBP2 antibody. Each image shows confocal microscopic images obtained from the center of each cochlear turn, and the number of CtBP2-positive puncta in each image is indicated. In the absence of NE, there was no significant difference in the number of CtBP2-positive puncta in IHC at each cochlear turn, between WT and TG mice. NE-induced significant decreases in the number of puncta of IHCs at Bs in TG mice compared to WT mice. Scale bar: 20 μm. **f** The cochleogram shows the number of CtBP2-positive puncta per IHC in each percentage distance from the apex of the cochlea at 8 weeks, with or without NE. In the post-NE group, a significant decrease at the distance of 80% from apex was observed in TG mice compared with WT mice (*n* = 7). **p = 0.0036 by two-way ANOVA with Bonferroni post-hoc testing. **g** The wave I amplitudes with 90 dB SPL at CK, 16 and 32 kHz at day 28 after NE in WT (*n* = 20) and TG (*n* = 24) mice were graphed. There were no significant differences between WT and TG mice.

**Fig. 3:**
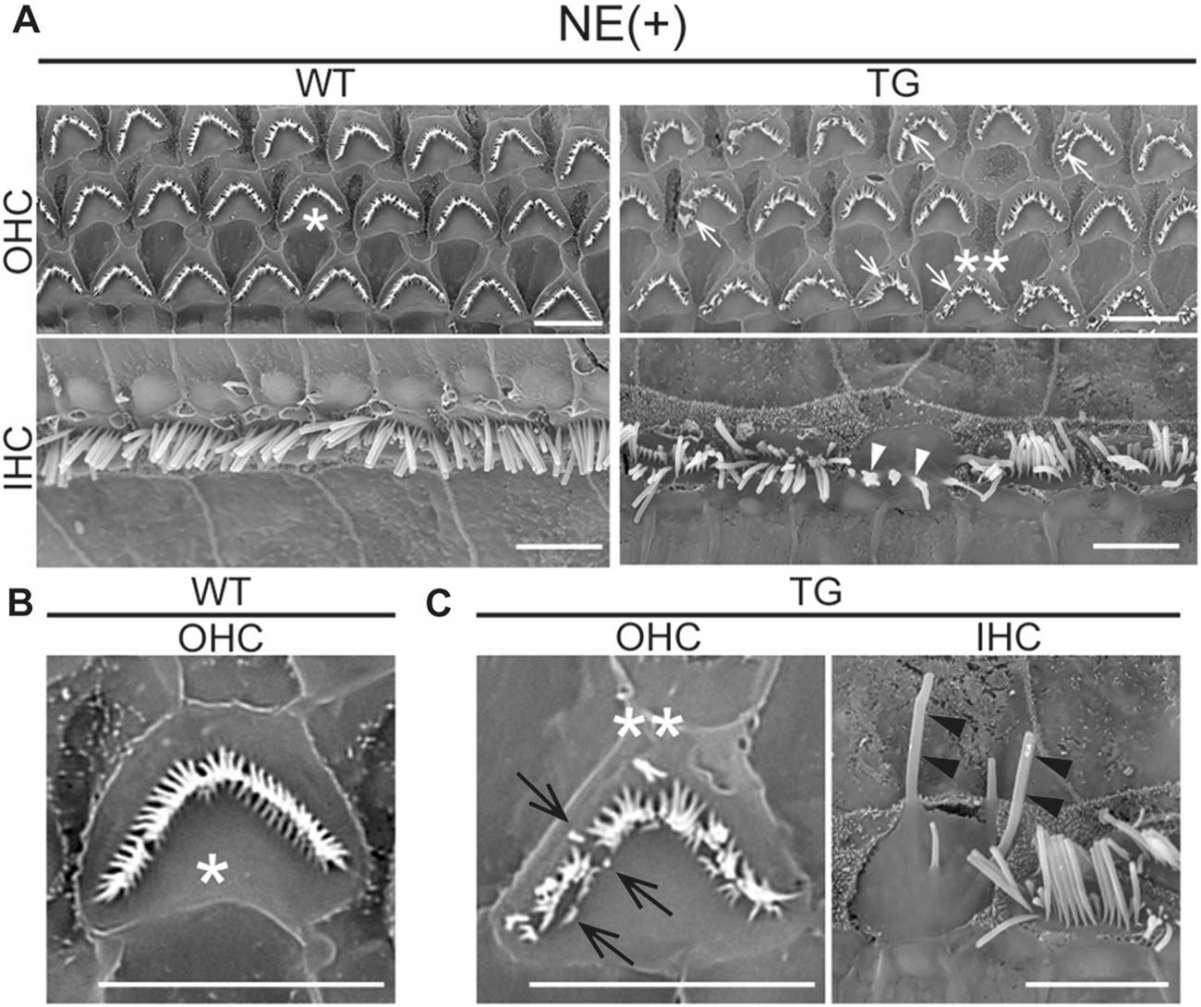
Ultrastructural changes of stereocilia in *DIA1*-TG mice after noise exposure. After noise exposure (NE) at the age of 4 weeks, the middle turns of cochleae in WT and *DIA1*^*TG/TG*^ (TG) mice were fixed at the age of 8 weeks (at day 28 after NE) for scanning electron microscopy (SEM). Note that HC loss after NE was not significant in WT or TG mice. Scale bars: 5 μm. **a** Low-magnification views of OHCs (upper panels) and IHCs (lower panels) of the cochleae are shown. In the TG mice, stereocilia were damaged in some of the OHCs and IHCs. Abnormally short and sparse (arrows), and fused (arrowheads) stereocilia are indicated. **b**, **c** High magnification views of a surviving OHC in WT (**b**) and surviving OHC and IHC in TG mice (**c**). Asterisks and double asterisks indicate the same OHCs, while the IHC is from the adjacent portion of the same sample. The short (arrows) and elongated stereocilia (arrowheads) are indicated.

AJCs are a major target of acoustic trauma^33^. To evaluate the vulnerability of cochlear HCs in TG mice, we examined the functional and morphological changes in the OC 28 days after NE (Fig. 2a). NE (100 dB for 2 h) has been shown to induce a moderate but reversible 40 dB temporary threshold shift (TTS) in auditory brainstem response (ABR) measurements^34^. We introduced a modified NE (105 dB for 1 h) to 4-week-old WT and TG mice, which produced 30 dB elevations (WT vs. TG; 32.4 ± 3.1 dB vs. 30.1 ± 2.1 dB, respectively) in ABR thresholds for all frequencies (Fig. 2a, b). Slight elevation of the ABR threshold by 32 kHz in TG mice relative to WT mice was observed at day 28 after NE (Fig. 2b). However, at the age of 8 weeks, no significant difference in ABR thresholds was observed between the NE and non-NE conditions at all frequencies in WT and TG mice (Fig. S3). With NE, slight OHC loss was observed in the basal turn of the cochlea of WT and TG mice; however, there were no significant differences in the number of residual HCs between WT and TG mice at day 28 (WT at apical, middle, and basal: 98.1 ± 0.87%, 99.0 ± 0.33%, and 83.8 ± 5.61% in OHCs, 97.3 ± 1.1%, 98.9 ± 0.94%, and 96.6 ± 1.24% in IHCs; TG at apical, middle, and basal: 98.2 ± 0.90%, 99.3 ± 0.21%, and 86.3 ± 4.4% in OHCs, 95.5 ± 1.28%, 100%, and 96.3 ± 0.70% in IHCs; Fig. 2c). This result was further confirmed by distortion product otoacoustic emission (DPOAE) measurements, which reflect OHC function^35^ (Fig. 2d). Conversely, the number of ribbon synapses in IHCs labeled by CtBP2 antibody was decreased at the basal turn of the cochlea in TG mice (75% along the cochlear length from the apex, at the center of the basal turn of the cochlea) compared to WT mice at day 28 with but not without NE (Fig. 2e). Detailed analysis using cochleograms revealed no significant difference in the number of CtBP2 punctae between WT and TG mice at 8 weeks of age; however, NE-induced decrements in the CtBP2 punctae at the basal turn of the cochlea (65–85% distance of the total cochlear length) were observed in the TG mice compared to WT mice (WT vs. TG at each % distance: 14.4 ± 0.91 vs. 10.0 ± 1.70 at 65%, *p* = 0.0789; 13.8 ± 1.21 vs. 10.3 ± 1.57 at 70%, *p* = 0.4186; 12.2 ± 0.88 vs. 7.8 ± 1.30 at 75%, *p* = 0.0867; 12.6 ± 0.93 vs. 6.3 ± 0.52 at 80%, where 42 kHz sound is mostly amplified^5^, **p = 0.0036; 11.5 ± 1.14 vs. 6.9 ± 0.88 at 85%, *p* = 0.0781; Fig. 2f). Wave I amplitudes measured at 32 kHz were not significantly lower in TG mice (Fig. 2g). Moreover, scanning electron microscopy (SEM) analysis revealed that ultrastructural changes, such as sparse, short, and fused stereocilia in HCs at the middle turn of the cochlea (Fig. 3a–c), where no significant HC loss occurred (Fig. 2c), were induced by NE in TG mice. Elongated stereocilia were also observed in TG mice after NE (Fig. 3c), although rarely. These data suggest that HCs in TG mice had subclinical vulnerability to acoustic trauma in stereocilia and ribbon synapses.

### AcGFP-DIA1(R1213X) localization in the OC and SG in *DIA1*-KI mice

**Fig. 4:**
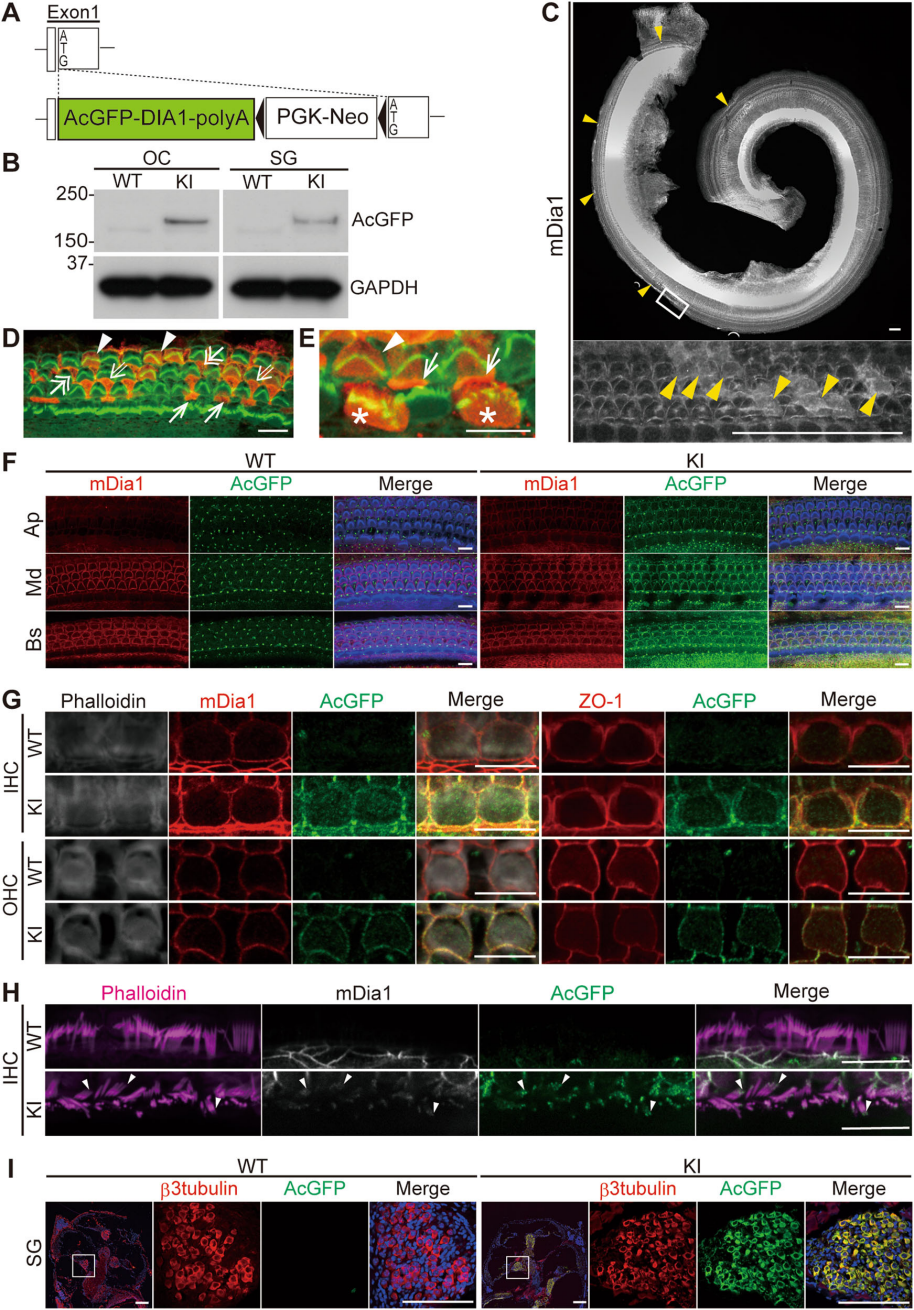
AcGFP-DIA1(R1213X) localization at AJCs and stereocilia in HCs and SGNs. **a** Illustration of the linearized expression cassette containing the *AcGFP-tagged DIA1(R1213X)*, *SV40 poly(A)* sequence, and *PGK-Neo* used for targeted injection instead of the exon1 of the *Dia1* gene, generating AcGFP-DIA1(R1213X) knock-in (*DIA1*^*KI*^) mice. **b** Lysates of the organ of Corti (OC) and the spiral ganglion (SG) were obtained from WT and *DIA1*^*KI/KI*^ (KI) mice at P5. Expression of AcGFP-DIA1(R1213X) was confirmed by immunoblotting using an AcGFP antibody. Comparative protein loading was confirmed by a GAPDH antibody. Uncropped images are shown in Fig. S1B. **c**–**e** Cochleae were obtained from the KI mice at P5, and immunostained using a mDia1 antibody and Alexa488-conjugate phalloidin. Arrowheads in low-magnification view show the dense regions of mDia1-positive cells. The lower panel shows the higher magnification view of the boxed region of the OC in the upper panel (**c**). Scale bars: 50 μm. **d**, **e** High magnification images of the OC (mDia1: red and phalloidin: green). Arrowheads, double-headed arrows, double-lined arrows, arrows, and asterisks show Dia1-positive outer hair cells (OHCs), Deiters’ cells (DCs), outer pillar cells (OPCs), inner pillar cells (IPCs), and inner hair cells (IHCs), respectively. Scale bars: 10 μm. **f** Whole-mount preparation of three turns of the OC were obtained from WT and KI mice at P7, and immunostained using mDia1 (red) and AcGFP (green) antibodies with Alexa405-conjugated phalloidin (blue). Confocal microscopic images focused at the apical junctional complex (AJC) plane are shown. Immunoreactivity of mDia1 and AcGFP are co-localized at the AJCs in the basal (Bs) and middle (Md) turns of the KI cochlea. Scale bar: 10 μm. Images with reduced background in the AcGFP staining are shown in Fig. S4. **g** Whole-mount preparation of the middle turn of the OC was obtained from WT and KI mice at P14. Immunostaining was performed using mDia1 (red) and AcGFP (green) antibodies with Alexa405-conjugated phalloidin (gray) or a ZO-1 (red) and AcGFP (green) antibodies. mDia1, AcGFP, and ZO-1 were co-localized at AJCs in OHCs and IHCs of the KI cochlea. Scale bars: 10 μm. **h** Immunostaining was performed using mDia1 (grey) and AcGFP (green) antibodies with Alexa405-conjugated phalloidin (magenta). AcGFP punctae were accumulated at the stereocilia tips of IHCs in the KI mice at P14. Scale bars: 10 μm. **i** Cryostat sections of cochleae at the plane of the SG from WT and KI mice were obtained at P14, and immunostained using β3-tubulin (red) and AcGFP (green) antibodies with DAPI (blue) using the TSA enhancing method. Low-magnification views of the cochlea, and high magnification views of the boxed regions at the middle turn of the cochlea, are indicated. Scale bars: 100 μm.

To further investigate which cells in the cochlea and which sites within the cells relate to constitutively active DIA1 mutant function and pathogenesis in DFNA1 patients, we generated AcGFP-DIA1(R1213X) knock-in (KI) (*DIA1*^*KI*^) mice (Fig. 4a, details about hearing function in this mouse line is being preparation for another publication). We confirmed AcGFP-DIA1(R1213X) expression in the OC and SG of *DIA1*^*KI/KI*^ (*DIA1*-KI) mice by IB using an AcGFP antibody (Takara Bio Inc., Kusatsu, Japan; Fig. 4b and Fig. S1).

Immunofluorescence staining of whole-mount cochleae at P5 in the KI mice using a mDia1 antibody revealed loco-regional mDia1-positive cells (Fig. 4c), involving OHCs, DCs, OPCs, IPCs, and IHCs (Fig. 4c–e). These expression patterns were similar to those in WT and TG mice (Fig. 1b, d, and Fig. S2). However, AcGFP fluorescence in the cochlea was too weak to detect, even with confocal laser scanning microscopy. Therefore, we enhanced the AcGFP fluorescence signal by immunofluorescence staining using an AcGFP antibody. The resulting immunofluorescence co-localized with mDia1 antibody immunoreactivity at the AJCs with a basal-to-apical gradient at P7 in the KI mice (Figs. 4f and S4). In the mature OC of KI mice at P14, the AcGFP signal was identified at AJCs in both IHC and OHC, and the junctional localization was confirmed by co-localization of mDia1 and a tight junction marker, ZO-1 (Fig. 4g). The junctional localization of the AcGFP-DIA1(R1213X) disappeared in WT mice (Fig. 4g). Moreover, AcGFP-DIA1(R1213X) accumulated at the stereocilia tips of IHCs at P14 in the KI mice (Fig. 4h). Some of these AcGFP signals were co-localized with mDia1 (Fig. 4h); however, neither AcGFP nor mDia1 signals at stereocilia tips were observed in WT mice. In addition, AcGFP-DIA1(R1213X) co-localized with β3-tubulin was detectable in SGNs in KI, but not WT, mice at P14 using TSA (Fig. 4i).

### Disruption of AJCs in MDCK^AcGFP-DIA1(R1213X)^ cells

**Fig. 5:**
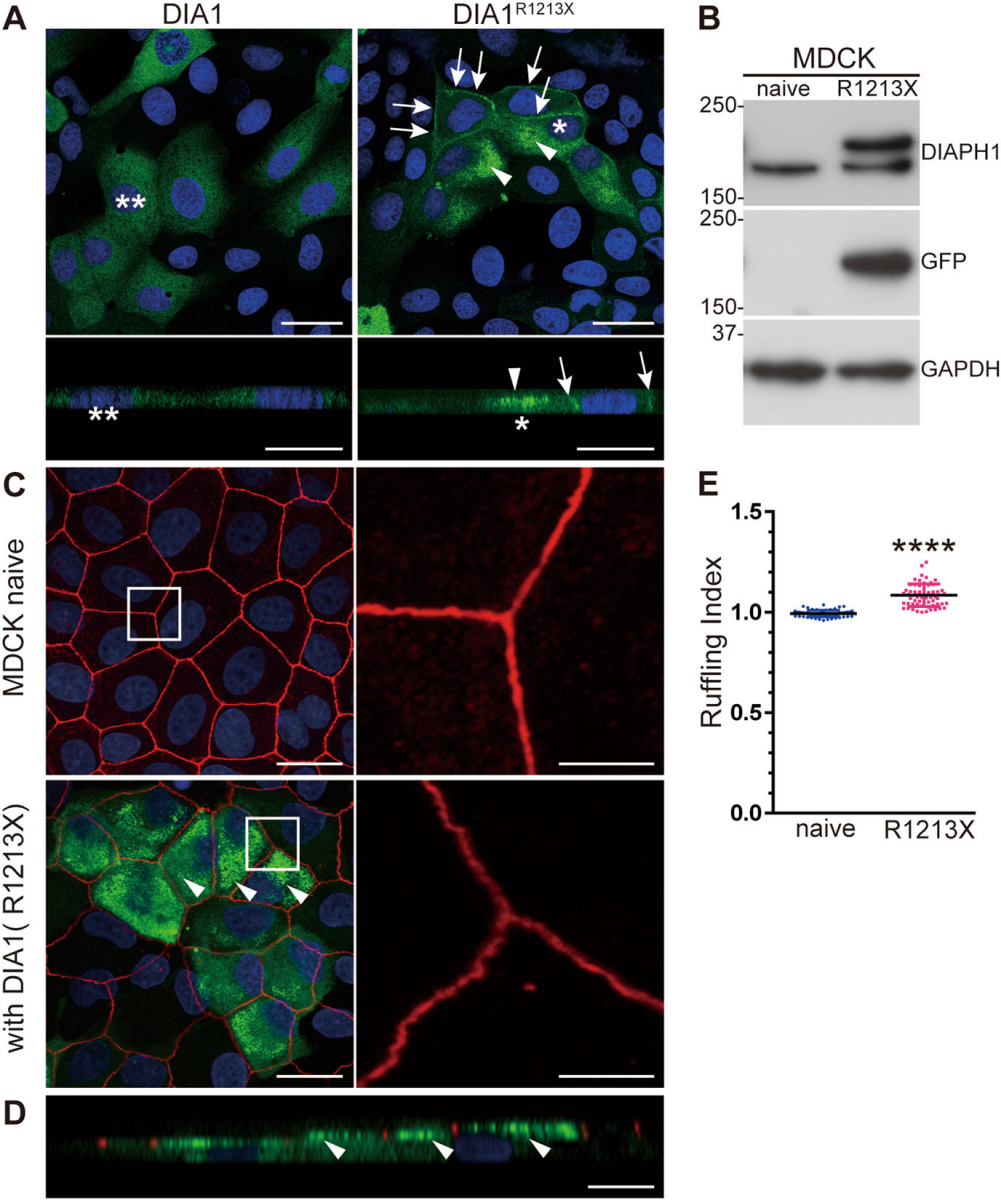
Abnormal tight junctions in MDCK^AcGFP-DIA1(R1213X)^ cells. **a** AcGFP-DIA1 or AcGFP-DIA1(R1213X) was transfected in MDCK cells. Thirty-two hours after transfection, cells were stained with DAPI (blue), and observed under a confocal microscope. Lower panels show lateral views of the upper panels. Asterisks and double asterisks indicate the same cells in the two different image views. In the right upper panel, AcGFP-DIA1(R1213X) was localized at the plasma membranes (arrows) and microvilli (arrowheads). Note the different localization pattern of AcGFP-DIA1(R1213X) compared to AcGFP-DIA1 in the left upper panel. Scale bar: 20 μm (upper panel), 10 μm (lower panel). **b** Immunoblotting using a green fluorescent protein (GFP; Cell Signaling Technology) antibody of naive MDCK and MDCK^AcGFP-DIA1(R1213X)^ cells revealed expression of AcGFP-DIA1(R1213X) (upper bands in the upper and middle panels) in addition to endogenous Dia1 (lower bands in the upper panel). Comparable protein loading is confirmed by a GAPDH antibody. Uncropped images are shown in Fig. S1C. **c** WT (naive) MDCK cells and MDCK cells with stable expression of AcGFP-DIA1(R1213X) (MDCK^AcGFP-DIA1(R1213X)^) were fixed, and stained using a ZO-1 antibody (red) with DAPI. Right panels show high magnification views of boxed regions in left panels. Tight junctions (TJs) labeled by ZO-1 antibody were ruffled in MDCK^AcGFP-DIA1(R1213X)^ cells. Scale bar: 20 μm (left panel), 5 μm (right panel). **d** The lateral view of MDCK^AcGFP-DIA1(R1213X)^ in panel **b**. Arrowheads indicate localization of AcGFP-DIA1(R1213X) at microvilli, but not TJs. Scale bar: 20 μm. **e** Graph showing the ruffling index in naive MDCK (*n* = 67) and MDCK^AcGFP-DIA1(R1213X)^ (*n* = 62) cells obtained three independent experiments (six dishes). The index was significantly increased in MDCK^AcGFP-DIA1(R1213X)^ cells. ****p < 0.0001 by unpaired Student’s *t-*test.

To further investigate the morphological changes caused by DIA1(R1213X) at the AJCs, we performed in vitro experiments using MDCK cells. In contrast to the cytoplasmic localization of WT AcGFP-DIA1, the AcGFP-tagged constitutively active mutant of DIA1, AcGFP-DIA1(R1213X), was localized at the plasma membranes and microvilli on the apical surface in transiently transfected MDCK cells (Fig. 5a). We established MDCK^AcGFP-DIA1(R1213X)^ cells, which stably expressed AcGFP-DIA1(R1213X). IB using a GFP antibody (Cell Signaling Technology, Danvers, MA, USA) revealed expression of the AcGFP-DIA1(R1213X) in MDCK^AcGFP-DIA1(R1213X)^ cells (Figs. 5b and S1). AcGFP-DIA1(R1213X) in MDCK^AcGFP-DIA1(R1213X)^ cells showed obscure junctional localization and stronger microvilli localization compared to those in MDCK cells with transient expression (Fig. 5c, d). The tight junctions of MDCK^AcGFP-DIA1(R1213X)^ cells stained by ZO-1 antibody were ruffled and irregular compared to those in naive MDCK cells (Fig. 5c, d). The ruffling index was significantly increased in MDCK^AcGFP-DIA1(R1213X)^ cells (Fig. 5e). These data suggest that Dia1 has a pivotal role in the development and maintenance of cell-cell junctions, and the constitutive activation of Dia1 may mis-localize it from tight junctions to microvilli on the apical surface.

### Disturbed ultrastructure of OHC in *DIA1*-TG mice

**Fig. 6:**
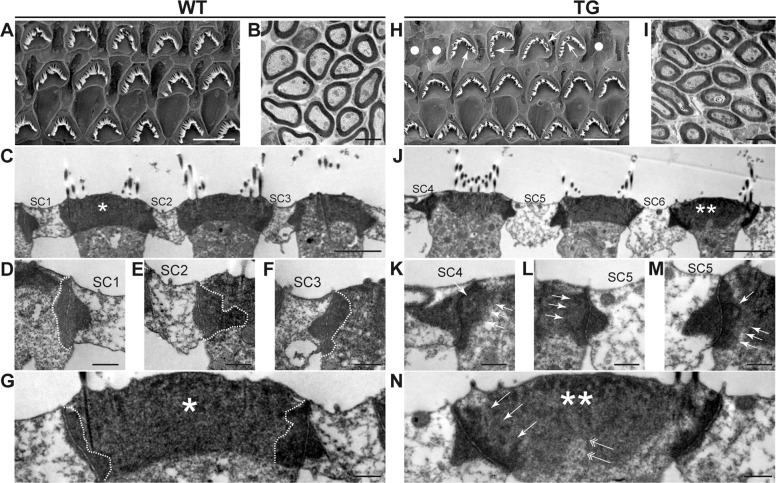
Ultrastructural analysis of the organ of Corti in 5-month-old *DIA1*-TG mice by transmission electron microscopy. Five-month-old WT (**a–g**) and *DIA1*^*TG/TG*^ (TG) (**h–n**) mice were fixed for scanning electron microscopy (SEM) and transmission electron microscopy (TEM). **a, h** SEM images of OHCs and supporting cells (SCs). OHC loss (dots) and abnormal, such as short and sparse (arrows), stereocilia in TG mice. **b**, **i** Peripheral nerves at the osseous spiral lamina at the basal turn of the cochlea in WT (**b**) and TG mice (**i**). **c–g, j–n** High magnification views of ultrathin section of OHCs at the distal portion of the middle turn (close to the basal turn) of the cochlea by TEM. Magnification views of apical junctional complexes (AJCs) and cuticular plates of OHCs in WT (**c**) and TG (**j**) mice were shown in **d**–**g** and **k**–**n**, respectively. Note the hazy (arrows in **k**–**m**), ruffled peri-junctional actin belts (arrows in **n**) and cuticular plates (double arrow in **n**) of OHCs in TG mice, compared to smooth actin belts (dotted lines) in WT mice (**d**–**g**). Asterisk and double asterisks indicate the same OHCs, respectively. Scale bar: 5 μm (**a**, **h**), 2 μm (**b**, **c**, **i**, **j**), and 500 nm (**d**–**g**, **k**–**n**).

We used SEM and transmission electron microscopy (TEM) to investigate the ultrastructural changes in OHCs at the distal portion of the middle turn of the cochlea, close to the basal turn, at 5 months of age. SEM images revealed OHC loss and abnormal stereocilia that were short and sparse in the TG mice (Fig. 6a, h). TEM analysis revealed that peripheral nerve fibers in the osseous spiral lamina were similar in WT and TG mice (Fig. 6b, i). Therefore, we focused on AJC changes in OHCs, since HC loss begins in the OHCs of TG mice^28^. In WT mice, OHCs and SCs were bordered by smooth arcuate-shaped apical junctional membranes and the underlying thick peri-junctional density of the circumferential actin belt (Fig. 6c–g). However, these actin dense regions were disturbed and became hazy or ruffled in TG mice (Fig. 6j–n). Moreover, the cuticular plate of OHCs in TG mice sometimes became irregular and obscure (Fig. 6n).

## Discussion

Dia1 has been well studied in many cell types; however, the expression pattern and physiological function of Dia1 in the cochlea remains unclear. Neuhaus et al. have reported that Dia1 was expressed in IPCs, SGNs, and oligodendrocytes at the transitional zone of the peripheral and central cochlear nerves^27^. In the present study, we identified novel Dia1 expression in different cell types in the OC, including DCs, OPCs, OHCs, and IHCs, using two Dia1 antibodies not used in the previous study, and in all four antibodies whose immunogens exist in the N-terminal regions of Dia1/DIA1. The weak and regional expression of Dia1 at P5 became ubiquitous, with basal-to apical progression, during cochlear maturation, and targeted specific regions of HCs at P14, such as the AJCs. We confirmed AJC localization of Dia1 in three different (WT, *DIA1*^*TG/TG*^, and *DIA*^*KI/KI*^) mice. *Drosophila* diaphanous reportedly plays a role in the development of the cochlear duct, and is essential for the regulation of junctional stability during morphogenesis^36,37^. The junctional localization of Dia1 and its role as a mediator for apical junctional integrity has been reported in many cell types^38–42^. Thus, we conclude that Dia1 is widely expressed in the OC and specifically localized to the AJCs during and following maturation of the OC.

We previously hypothesized that the constitutively active mutant of DIA1 enhances actin polymerization not only at stereocilia but also at AJCs, since we observed short- and sparse-form dominant phenotypes of stereocilia and AJC deformities between HCs and SCs^28^. In the present study, we demonstrate that the constitutively active mutant of DIA1 was localized in the AJCs and stereocilia tips of the KI mice, where actin turnover occurs^43^. The deformity of AJCs in TG mice and MDCK^AcGFP-DIA1(R1213X)^ cells further supports this hypothesis. Furthermore, another constitutively active mutant of *Xenopus laevis* Dia1 with mutation in the C-terminal DAD causing p.F1192A, has been shown to localize to the tight junction and induce its rippled shape^42^. Since the cochleae of *Dia1*-knockout mice are morphologically and functionally normal^28^, the constitutively active mutants of DIA1, p.R1213X/p.R1204X, work at the AJCs in a gain-of-function manner, leading to loss of cell polarity and integrity of the OC and HCs. Additionally, the membrane and microvilli localization of the active mutant in MDCK cells, with transient overexpression, suggested that the active mutant also contributes to organizing the actin cytoskeleton in stereocilia. Indeed, NE-induced elongated stereocilia in damaged IHCs of TG mice.

Conversely, in stable MDCK^AcGFP-DIA1(R1213X)^ cells, we were unable to recapitulate the cochlear AJC localization of DIA1(R1213X) observed in TG and KI mice. We established several MDCK cell lines with stable expression of AcGFP-DIA1(R1213X); however, the active mutant showed obscure junctional localization in cell lines in which it was highly expressed (e.g., Fig. 5b). This discrepancy may be explained by the possibility that cells with excessive expression/activity of Dia1 at their AJCs undergo cell death. Thus, progressive hearing loss in TG mice, as a DFNA1 mouse model, is likely induced by disorganized actin polymerization in AJCs and stereocilia.

An inability to repair damages to HCs, resulting from normal daily sound exposure in life, might explain hearing loss in DFNA1 patients^23^. Involvement of the RhoA-mDia1 signaling axis was reported with actin re-organization after NE in the cochlea^44,45^. In NE and aging, the ribbon synapse is a primary and sensitive target for underlying damage to neurons and HCs^46^. Furthermore, electrophysiological studies revealed that cochlear synaptopathy was involved in IHC malfunction induced by acoustic trauma^47^. We found cytoskeletal and synaptic vulnerabilities to moderate NE in TG mice. Considering the multifocal ultrastructural changes of HCs in aged TG mice, constitutive activation of Dia1 may widely affect actin remodeling pathways in HCs. Morphological defects at the cuticular plates and stereocilia in *DIA3*-overexpressing mice may support our results and theories^48^. Moreover, polymerized actin plays a role in the formation of membrane invagination at synapses^49^, as well as Dia1 regulated synaptic vesicle endocytosis at the presynaptic active zone^50^. Taken together, excessive and disorganized mechanosensitive actin assembly may be triggered by NE in DFNA1 patients, thereby leading to HC damage, stereocilia deformities, and ribbon synapse decrement. Since DFNA1 patients may have subclinical vulnerability in HCs induced by constitutive activation of DIA1, avoiding NE and pharmacological interventions to reduce disorganized actin polymerization may mitigate progressive hearing loss in DFNA1 patients.

Considering the central nervous system phenotype of the loss-of-function mutant of Dia1^51,52^, postsynaptic factors could underlie the synaptic vulnerability of TG mice. We demonstrated Dia1 expression in SGNs using KI and TG mice. However, no significant ultrastructural changes in the cochlear nerve at the osseous spiral lamina were identified in TG mice at 5 months, when destruction of AJCs was observed^28^. Moreover, contrary to a previous report^27^, we did not find endogenous Dia1 and active DIA1 mutant signals at the transitional zone of the cochlear nerve. Thus, lesions caused by the constitutively active mutants of DIA1 are most likely at the OC. However, the physio-pathological role of Dia1 in SGNs, and the relevance to cochlear synaptopathy induced by NE in TG mice, remains unknown. Further studies on neuronal vulnerability in DFNA1 are required.

In summary, to the best of our knowledge, we report, for the first time, the expression patterns of endogenous Dia1 and the constitutively active mutants of DIA1 in the cochlea. Dia1 is localized to the AJCs between HCs and SCs during and after maturation of the OC, and contributes to establishing HC integrity. Constitutive activation of DIA1 results in disorganized cytoskeletal turnover, leading to HC vulnerability and cochlear synaptopathy. Our findings may provide new therapeutic targets for the prevention of the HC degeneration and progressive hearing loss associated with DFNA1.

## Materials and methods

### Plasmids

Human DIA1 was amplified through PCR and cloned into pAcGFP (C1) (Takara Bio), to create a plasmid we named AcGFP-DIA1. R1213X mutant, which terminates at 1213 aa due to the 3637 C > T mutation located in exon 27, was introduced into AcGFP-DIA1 using a QuickChange Lightning Site‐Directed Mutagenesis kit (Agilent Technologies Inc., Santa Clara, CA, USA). DIA1(R1204X), which we previously reported^28^ DIA1(R1213X) has the same mutation in the DAD, but DIA1(R1204X) and DIA1(R1213X) originate from *DIA1-2* and *DIA1-1* alternative splicing variants that differ in one additional or missing exon, consisting of 9 aa.

### Animals

This study was approved by the Institutional Animal Care and Use Committees (reference: 26-03-05) and carried out in accordance with the Animal Experimentation Regulation of Kobe University.

We previously described^28^ a transgenic mouse model of DFNA1 expressing 3xFLAG-tagged DIA1(R1204X) mutant (*DIA1*-TG or *DIA1*^*TG/+*^), in which four transgenic copies were integrated into the heterozygous TG mice and the ratio of mutant DIA1 protein to endogenous Dia1 protein in the cochlea is ~1:1 in *DIA1*^*TG/+*^ mice.

We generated a knock-in (KI) mouse model of DFNA1 expressing AcGFP-tagged DIA1(R1213X) mutant (*DIA1*-KI or *DIA1*^*KI/+*^). The targeting vector was constructed by cloning the cDNA of human *DIA1-1* into the Xho1/Sal1 site of the pAcGFP-C1 using an In-Fusion HD Cloning kit (Takara Bio), and *DIA1*(R1213X) was made using a QuickChange Lightning Site-Directed Mutagenesis kit (Agilent Technologies). After confirming the identity of the plasmid by sequencing and expression of AcGFP-DIA1(R1213X) in HEK293 cells by IB, the *AcGFP-DIA1(R1213X)-SV40 polyA-FRT-PGK-Neo-FRT* construct was made and introduced into embryonic stem (ES) cells with a C57BL/6 background by electroporation, targeting the site just before ATG (initiation site) of the exon1 of Dia1. Homologous recombinant ES cell clones were identified by PCR and southern blotting to generate chimeric embryos, and were transferred to recipient C57BL/6 mice (Unitech, Kashiwa, Japan). Founder (F1) mice were screened by PCR using the following primer pairs: 5′-AGGATGACGGCAACTACAAGTC-3′ (in the AcGFP) and 5′-CCTTCTTAATTCTCATGCTGGTAAA-3′ (in DIA1(R1213X): coding nucleotides 178–202), 5′-TTCACAATTTTCGGAATATGTTTTT-3′ (in DIA1(R1213X): coding nucleotides 3413–3437) and 5′-CTTCCTCGTGCTTTACGGTATC-3′ (in the neomycin selection cassette). Offspring were genotyped by PCR using the following primer pair: 5′-CGTAGACAAGGGGTCACTTG-3′ (in the AcGFP) and 5′-TTAGATTTGCCGCCGTCGCC-3′ (in the DIA1(R1213X): coding nucleotides 92–111). F2 and later generations of the homozygous KI mice (*DIA1*^*KI/KI*^) were used for subsequent analyses.

Mice were group-housed in the animal facility of Kobe University under specific pathogen-free conditions using an individually ventilated care system (Tecniplast Japan, Tokyo, Japan) and were maintained on a 12-h light and 10-h dark cycle at 23 ± 2 °C and 50 ± 10% humidity, with food and water ad libitum. All mice were identified by numbered ear tags. Both male and female mice were used in the analyses unless otherwise indicated (mice younger than 1 week were not differentiated based on sex). Age-matched wild type (WT) siblings were used as controls. Mice from the control group were always treated and assessed first, followed by the experimental group. No randomization was performed.

### Immunoblotting

Murine cochleae and modioli without cochlear nerves (hereafter SG) were dissected at P5 and lysed by sonication in homogenizing buffer containing Protease Inhibitor Cocktail (Nacalai Tesque, Kyoto, Japan), as described previously^53,54^. Total cell lysates were centrifuged at 12,000 × *g* for 5 min at 4 °C, and the supernatants were subjected to sodium dodecyl sulfate-polyacrylamide gel electrophoresis (SDS-PAGE) followed by IB for 2 h at 23 °C using primary antibodies diluted in phosphate-buffered saline (PBS) and 0.03% TritonX-100 (TX) containing 0.5% bovine serum albumin (BSA). The bound primary antibodies were detected with secondary antibody-HRP conjugates using the enhanced chemiluminescence (ECL) detection system (Bio-Rad Laboratories, Hercules, CA, USA).

### Immunohistochemistry

To analyze whole-mount preparations of the OC, cochleae were dissected from the temporal bones, and fixed with 2–4% paraformaldehyde (PFA) in 0.1 M phosphate buffer (PB; pH 7.4) for 30 min at 23 °C. In cases of mice older than P14, cochleae were decalcified in 0.12 M ethylenediaminetetraacetic acid (EDTA) at 23 °C for 48 h and then dissected into four pieces (apical, middle, basal turn, and hook). Tissues were blocked with 5% BSA in PBS and 0.3% TX for 1 h followed by incubation with primary antibodies with 1% BSA in PBS and 0.03% TX for 2 h at 23 °C. Tissues were rinsed in PBS and 0.03% TX, and incubated for 30 min at 23 °C in species-appropriate secondary antibodies.

To analyze paraffin-embedded cross-sections of cochleae at P8, cochleae were harvested and fixed with 4% PFA in 0.1 M PB for 30 min, followed by decalcification in 0.12 M EDTA for 48 h at 23 °C. Decalcified cochleae were embedded into paraffin blocks and cut into 12-μm slices on a Leica RM2125 RT (Leica Biosystems, Nussloch, Germany). Sections were immunostained after deparaffinization, as described previously^54^. To analyze cryostat sections of cochleae at P7–14, cochleae were fixed with 2% PFA in 0.1 M PB for 30 min, followed by decalcification in 0.12 M EDTA for 2 h at 23 °C and dehydration in 30% sucrose at 4 °C overnight. To make thin sections of the hard tissue, we introduced SCEM compound (SECTION-LAB, Hiroshima, Japan) and adhesive film (Cryofilm type IIC9, SECTION-LAB) according to Kawamoto’s film method, described previously^55,56^. Samples were embedded in the SCEM compound and the cut surface was covered with the adhesive film, then 10-μm-thick frozen sections were created using a cryostat (Leica CM1860; Leica Biosystems) with a carbide metal disposable blade (Leica TC-65; Leica Biosystems) at −20 °C to −23 °C. Sections were mounted on slide glass and kept at −30 °C until use. The sections were permeabilized in PBS and 0.3% TX, and blocked with 0.5% TSA blocking reagent (PerkinElmer, Courtaboeuf, France) in 0.1 M TRIS-HCl, pH 7.5, and 0.15 M NaCl (TN blocking buffer [TNB]) for 1 h at 23 °C, followed by incubation with primary antibodies in TNB for 2 h at 23 °C. The sections were rinsed briefly in PBS and 0.03% TX, then incubated with HRP- and Alexa 488(546)-conjugated secondary antibodies in TNB for 30 min at 23 °C. These antibodies were subjected to centrifugation at 15,000 rpm before use, to reduce nonspecific staining. The resulting sections were rinsed in PBS and 0.03% TX, followed by signal amplification using the TSA fluorescence system (PerkinElmer) for 5–10 min at 23 °C in accordance with a company protocol. The nuclei of SGs were counterstained with 4′6-diamidino-2-phenylindole (DAPI).

To analyze MDCK cells, samples on the glass-bottom dish were fixed by 4% PFA in 0.1 M PB. After permeabilization in PBS and 0.3% TX with 0.5% fat-free BSA, fixed cells were incubated with primary antibodies in PBS and 0.03% TX, followed by Alexa-conjugated secondary antibodies, and counterstained with DAPI.

Stained tissues were mounted in ProLong Antifade (Thermo Fisher Scientific, Carlsbad, CA, USA) with a coverslip and imaged at low magnification (X5 objective) using a fluorescent microscope (Keyence, Osaka, Japan), and at high magnification using a confocal microscope (LSM700; Carl Zeiss, Jena, Germany). To identify immunostained cell types in the OC more clearly, reconstructed lateral projections were obtained after volume image processing of individual image stacked in 0.40 μm intervals using ZEN software (Carl Zeiss).

### Antibodies

The following specific antibodies were used (monoclonal unless indicated): CtBP2 (BD Biosciences, Cat# 612044, RRID:AB_399431; 1:200); GFP polyclonal raised against AcGFP (Takara Bio, Cat# 632592, RRID:AB_2336883; 1:200 [or 1:5000–10,000 when using TSA] for Immunohistochemistry (IH) and 1:1000 for IB); GFP (Cell Signaling Technology, Cat# 2956, RRID:AB_1196615; 1:1000 for IB); mDia1 raised against mouse Dia1 (aa 41–153) (BD Bioscience, Cat# 610848, RRID:AB_398167; 1:200 for IH and 1:1000 for IB); DIAPH1 (DIA1) raised against human DIA1 (aa 1–100) (Abcam, Cat# ab96784, RRID:AB_10680247; 1:5000 to 10,000 for IH using TSA and 1:2000 for IB); ZO-1 (Thermo Fisher Scientific, RRID:AB_2533147; 1:200); FLAG (clone M2, Sigma-Aldrich, RRID:AB_262044; 1:1000); *β*3-tubulin (Abcam, RRID:AB_2256751 and RRID:AB_444319 [polyclonal]; 1:300 for IH and 1:2000 for IB); and Myosin 7a polyclonal (Proteus Biosciences, Cat# 25–6790, RRID:AB_10015251; 1:500). Between mouse Dia1 and human DIA1, there are seven aa differences in aa 41–153 and six aa differences in aa 1–100. The HRP-conjugated anti-glyceraldehyde 3-phosphate dehydrogenase (GAPDH) (RRID:AB_10699462; 1:2000) antibody was previously described^28^. Alexa Fluor 564-conjugated anti-mouse IgG1 (RRID:AB_2535765; 1:500), Alexa Fluor 488-conjugated anti-rabbit IgG (RRID:AB_143165; 1:500), Alexa Fluor 405‐conjugated phalloidin (RRID:AB_2315147; 1:500), and HRP‐conjugated secondary (RRID:AB_2337943 and RRID:AB_2313567; 1:10,000 for IB and 1:200 for IH using TSA) antibodies were obtained from Thermo Fisher Scientific and Jackson Immuno Research Laboratories (West Grove, PA, USA). Validation data for each antibody is available from the respective companies.

### ABR and DPOAE measurements

To assess hearing, *DIA1*^*TG/TG*^ mice and littermate control WT mice were tested by ABR and DPOAE measurements at the age of 4 and 8 weeks, as described previously^28^. Briefly, mice were anesthetized with an intraperitoneal injection of an anesthetic cocktail (medetomidine 0.3 mg/kg, midazolam 4.0 mg/kg, and butorphanol 5.0 mg/kg), and placed on a heating pad to maintain body temperature. BioSigRP Software and the TDT System 3 (Tucker-Davis Technologies, Alachua, FL, USA) generated all stimuli and recorded all responses. ABR waveforms were recorded for 12.8 ms at a sampling rate of 40,000 Hz by using a 50–5000-Hz passband, and averaged 500 responses at each SPL. Clicks and 8 to 32 kHz tone bursts were presented in 5 dB steps from 90 dB SPL below threshold up to 10 dB SPL. Hearing threshold was defined as the lowest sound intensity level at which a recognizable or reproducible wave III was observed. The analysis was carried out offline in BioSigRP on traces with visible waves. The amplitude of wave I by clicks, and torn burst stimulus at 16 and 32 kHz with 90 dB SPL was determined as the time from the onset of the stimulus to the peak, while amplitude was measured by taking the mean of the ΔV of the upward and downward slopes of the peak.

DPOAEs were measured by using commercial instrumentation (HearIDTM Auditory Diagnostis System; Mimosa Acoustics, Champaign, IL, USA) combined with CUBeDIS II v2.40 (Etymotic Research; Elk Grove Village, IL, USA) software. For DPOAEs, the cubic distortion product 2f1-f2 was elicited in response to two primary sine wave tones, *f*1 and *f*2 (frequency ratio *f2*/*f1* = 1.2), where *f2* varied from 4 to 24 kHz in half-octave steps, with sound pressure levels of 65 and 55 dB SPL, respectively.

### Noise exposure

Four-week-old *DIA1*^*TG/TG*^ mice and littermate control WT mice were anesthetized with the anesthetic cocktail and exposed to octave-band-noise, centered at 8 kHz, for 1 h at 105 dB SPL in a sound chamber, as described previously^57^. Each animal was placed in the center of the chamber. To assess an ABR threshold shift after NE, the mice were measured for ABR at pre- and post-NE day 0 and 28, as shown in Fig. 2a.

### Pre-synaptic ribbons and hair cell count

Immunostained cochleae were imaged at 300 μm-spaced regions (approximately 5% distance of the average total cochlear length of C57BL/CBA mice^58^) from the apex using a confocal microscope (LSM700) with an oil-immersion x63 objective (1.4 numerical aperture). Each image contained 10–12 adjacent IHCs, stacked in 0.40 μm intervals, with the span adjusted to include all synaptic ribbons. Maximum intensity projection images were obtained from each z-stack image using ZEN software (Carl Zeiss). Pre-synaptic ribbons in IHCs were counted using Image J 1.43 u (available at http://rsb.info.nih.gov/ij; developed by Wayne Rasband, National Institutes of Health, Bethesda, MD, USA) and the average number of ribbons per IHC was then calculated at each region of the cochlea. For residual IHC and OHC counts at day 28 post-NE, HCs were counterstained with phalloidin. We acquired images at five different regions in the apical, middle, and basal cochlear turns by using the same apparatus, and manually counted HCs with stereocilia. Average residual HCs were then calculated in each turn respectively.

### Cell culture

MDCK cells^28^ and HEK293 cells (ATCC, RRID: CVCL_0045) were grown in Dulbecco’s Modified Eagle Medium (DMEM) supplemented with 10% FBS (Thermo Fisher Scientific) in a 5% CO_2_ humidified incubator at 37 °C. For transient expression experiments, 1.0 × 10^6^ MDCK cells were collected and transfected with AcGFP-DIA1 or AcGFP-DIA1(R1213X) by electroporation (NEPA21; NEPAGENE Co., Ltd, Ichikawa, Japan). The transfected cells were spread on a 3.5-cm glass-bottom dish (MatTek, Ashland, USA) and grown for 32 h. After fixation with 4% PFA in 0.1 M PB, the resulting cells were stained with the ZO-1 antibody and DAPI. MDCK^AcGFP-DIA1(R1213X)^ with stable expression of AcGFP-DIA1(R1213X) were established using electroporation followed by G418 selection (0.5 mg/ml; FUJIFILM Wako, Osaka, Japan).

### Quantification of the irregularity of tight junctions in MDCK cells

Stacked confocal microscopy images at the level of tight junctions were obtained using a confocal microscope (LSM700) with an oil-immersion ×63 objective. Five areas were randomly selected and captured for one sample. Measurement of the perimeter in a single cell, immune-labeled with the ZO-1 antibody, was performed using Image J 1.43 u, and we then acquired the sum of the straight lines connecting two vertices in the same cell. The ratio of actual measurement to the sum of the straight lines was calculated and defined as the ruffling index.

### Ultrastructural analysis using SEM and TEM

Freshly dissected cochleae of WT and *DIA1*^*TG/TG*^ mice were fixed with 2% PFA and 2.5% glutaraldehyde (GA) in 0.1 M PB for 2 h, followed by a post-fixation with 1% osmium tetroxide (OsO_4_) in H_2_O for 1 h at room temperature. For SEM analysis^28^, tissues were dehydrated through a graded ethanol series, followed by tert-Butyl alcohol, and dried in a freeze dryer (Eyela FD-5N; Tokyo Rikakikai Co., Ltd., Tokyo, Japan). Dried tissues, mounted on stages, were sputter-coated with gold in an Ion Sputter E-1010 (High-Technologies, Tokyo, Japan), and observed using a TM3030Plus SEM (Hitachi High-Technologies). For TEM analysis^54^, after block staining with 30% EM Stainer (Nisshin EM Co., Ltd., Japan) for 30 min at 23 °C, tissues were dehydrated through a graded ethanol series, embedded in Spur Low-Viscosity Embedding Media (Polysciences Inc., Warrington, PA, USA) and polymerized at 70 °C for 8 h. Ultrathin sections (thickness ∼100 nm) were cut on an ultramicrotome (EM-UC7; Leica Microsystems, Wetzlar, Germany), placed on copper grids, and stained with 30% EM Stainer for 20 min and lead citrate for 5 min at 23 °C. The grids were examined on a Hitachi H-7100 (Hitachi High-Technologies) electron microscope at 75–110 kV.

### Statistical analysis

Blinded data analysis was performed by two otologists or two scientists. All data are presented as mean ± standard errors of the mean. Statistical analyses were performed using Prism 6.0 software (GraphPad Software Inc., La Jolla, CA, USA), using an unpaired two-tailed Student’s *t-*test or two-way analysis of variance (ANOVA) followed by Bonferroni post-hoc test of pairwise group differences. Differences between groups were considered statistically significant when *p < 0.05, **p < 0.01, ***p < 0.001 or ****p < 0.0001. Sample size calculation was not performed.

## Supplementary information

Supplementary Information: legends of supplementary figures

Supplementary Figure S1

Supplementary Figure S2

Supplementary Figure S3

Supplementary Figure S4
