# Supplementary material for: The integrity of cochlear hair cells is established and maintained through the localization of Dia1 at apical junctional complexes and stereocilia
Source: Cell Death Dis. 2020 Jul 16;11(7):536. doi: 10.1038/s41419-020-02743-z (PMC7366933; doi:10.1038/s41419-020-02743-z)
Supplement: Supplementary file 1 — Supplementary Information: legends of supplementary figures [file 41419_2020_2743_MOESM1_ESM.docx]

**Supplementary Information: legends of supplementary figures (Figs S1–4).
Supplementary Fig. 1: Uncropped images of immunoblotting.**

Uncropped immunoblotting images of Figs. 1A (**A**), 4B (**B**), and 5B (**C**) are shown. Black lines in images indicate positions cut for immunoblotting using indicated primary antibodies. Red boxes indicate regions cropped and used for figures in the main text. Exposure times to films are shown below the panels.

(**A**) The left membranes were immunoblotted using DIAPH1, β3-tubulin, and GAPDH antibodies, and the right membranes was immunoblotted using FLAG and GAPDH antibodies. The same lysates and same amount of proteins were loaded in two membranes.

(**B**) The membranes were immunoblotted using an AcGFP or GAPDH antibody. The right and left panel were exposed to films for 1 sec and 8 sec, respectively.

(**C**) The membranes were immunoblotted using a DIAPH1 (left) or GFP (right) antibody with GADPH antibody. The same lysates and protein amounts were loaded in two membranes.

**Supplementary Fig. 2: mDia1-positive supporting cells in mid-modiolar sections of the cochlea and a reconstructed lateral projection view of the OC in *DIA1*-TG mice.**

Paraffin embedded mid-modiolar sections (12 μm, **A–C**) at P8 and whole-mount preparations of the organ of Corti (OC) at P5 (**D–F**) were obtained from the *DIA1^TG/TG^* (TG) mice. Immunostaining was performed using a mDia1 (red) and Myosin 7a (green) antibodies with DAPI (blue). Inner hair cell: IHC, outer hair cell: OHC.

(**A**) Low magnification images at the mid-modiolus (Mo) of sections were obtained using confocal fluorescence microscopy. mDia1-positive cells (red) were observed in the basal and middle cochlear turn (indicated by boxes). Hair cells (HC) were immunostained with a Myosin 7a antibody (green). Scale bar: 100 μm.

(**B** and **C**) High magnification images of the boxed regions in A. Double-headed arrows and double-lined arrows indicate the mDia1-positive (red) Deiters' cell (DC) and outer pillar cells (OPCs), respectively. Scale bars: 10 μm.

(**D–F**) Reconstructed lateral projection views of the OC at the basal cochlear turn were obtained using confocal microscopy. Double-headed arrows, double-lined arrows, and arrows indicate the mDia1-positive (red) DC, OPC, and inner pillar cell (IPC), respectively. Scale bars: 10 μm.

**Supplementary Fig. 3: Comparison of auditory brainstem response thresholds at 8 weeks with or without noise exposure after 4 weeks in WT and *DIA1-*TG mice.**

Auditory brainstem response (ABR) thresholds (dB SPL) at click (CK), 8, 16, 24, and 32 kHz in WT and *DIA1^TG/TG^* (TG) mice were measured at the age of 8 weeks (8W), with (*n*=14) or without NE (*n*=8) after 4 weeks (4W). There were no significant differences at any frequency among the four conditions (by two-way ANOVA followed Bonferroni post-hoc test). The data of the groups with NE are the same as shown in Fig. 2B.

**Supplementary Fig. 4: AcGFP staining with reduced background in the OC from WT and *DIA1*-KI mice.**

Whole-mount preparations of three turns of the OC were obtained from WT and *DIA1^KI/KI^* (KI) mice at P7, and immunostained using mDia1 (red) and AcGFP (green or grey) antibodies with Alexa405-conjugated phalloidin (blue). Confocal microscopic images focused at the apical junctional complex (AJC) plane were obtained using a low detector gain (the same image acquisition parameters were used between WT and KI mice) to eliminate background signals of AcGFP. Arrowheads indicate immunoreactivity of AcGFP at the AJCs in the apical (Ap), middle (Md), and basal (Bs) turns of the KI cochlea. Note the almost completely diminished AcGFP signal in WT cochlea. Scale bars: 10 μm.
