# Supplementary figures and images for: The integrity of cochlear hair cells is established and maintained through the localization of Dia1 at apical junctional complexes and stereocilia
Source: Cell Death Dis. 2020 Jul 16;11(7):536. doi: 10.1038/s41419-020-02743-z (PMC7366933; doi:10.1038/s41419-020-02743-z)

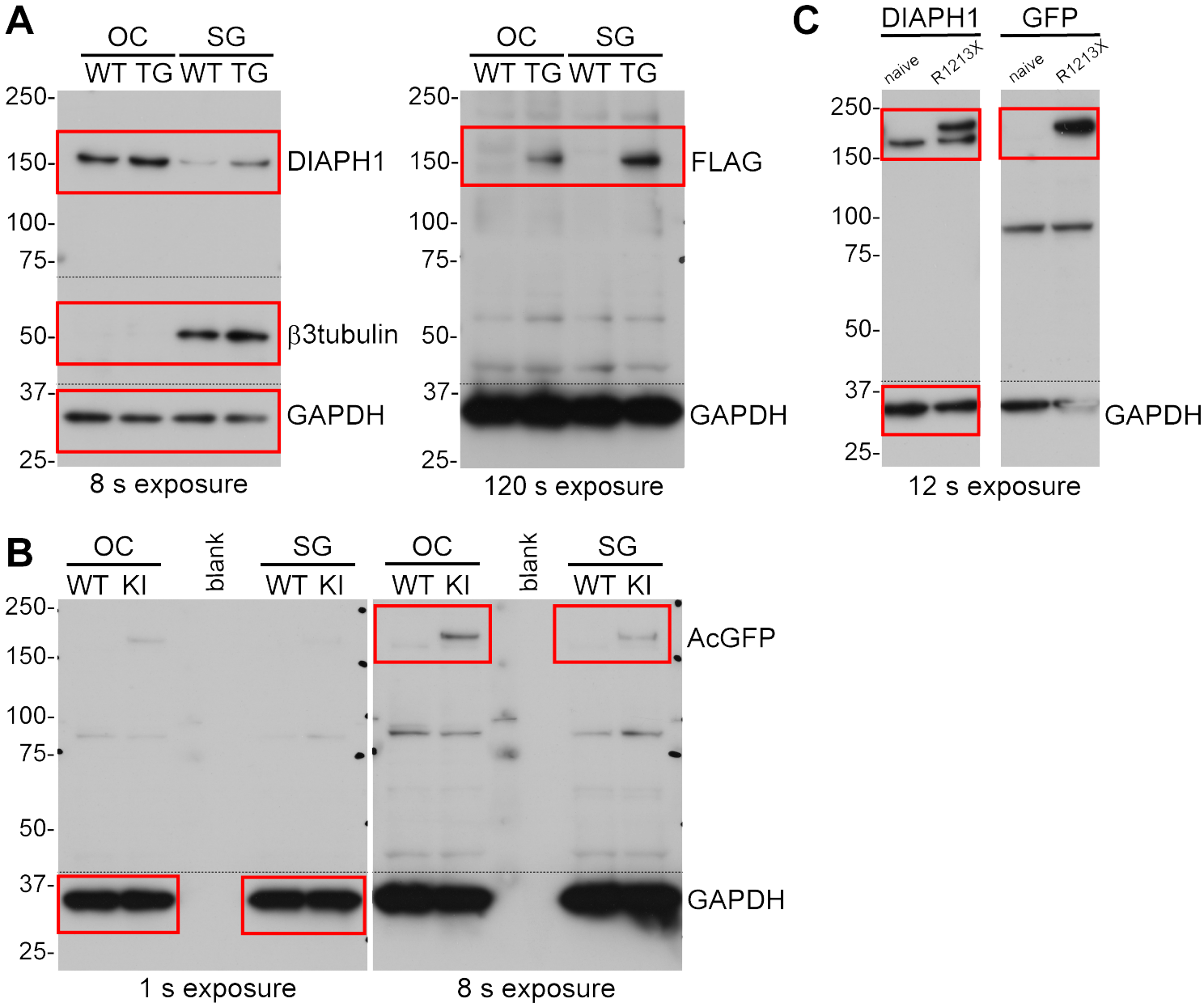

Supplement: Supplementary file 2 — Supplementary Figure S1 [file 41419_2020_2743_MOESM2_ESM.tif]

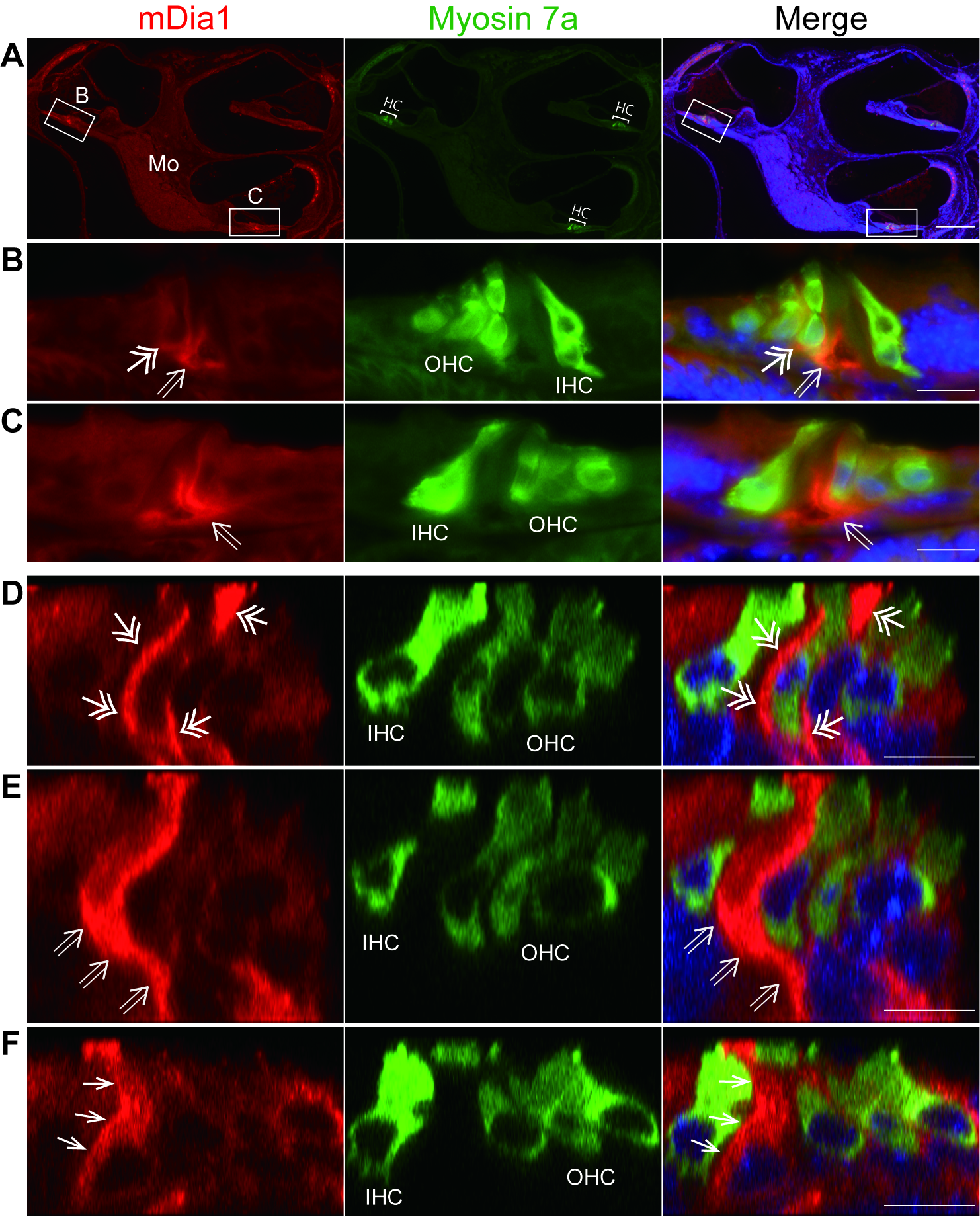

Supplement: Supplementary file 3 — Supplementary Figure S2 [file 41419_2020_2743_MOESM3_ESM.tif]

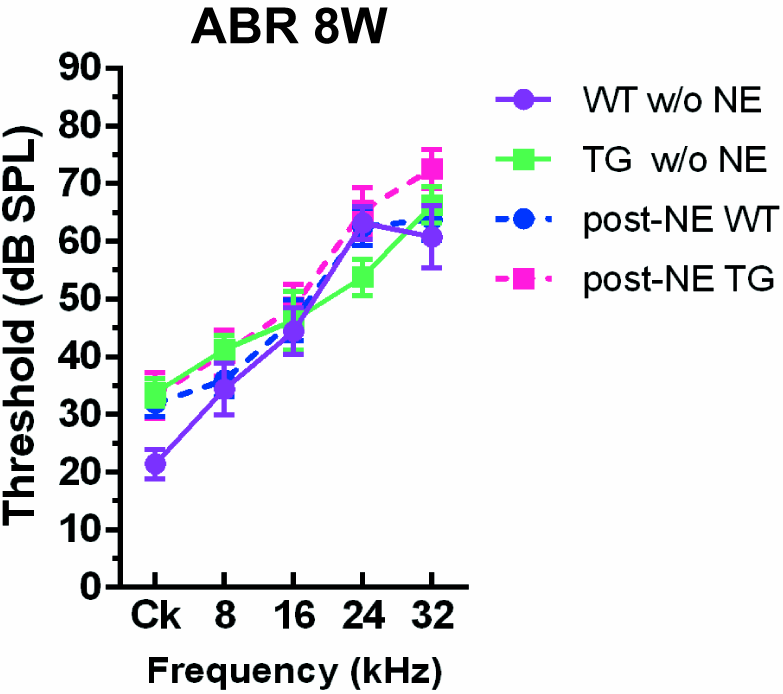

Supplement: Supplementary file 4 — Supplementary Figure S3 [file 41419_2020_2743_MOESM4_ESM.tif]

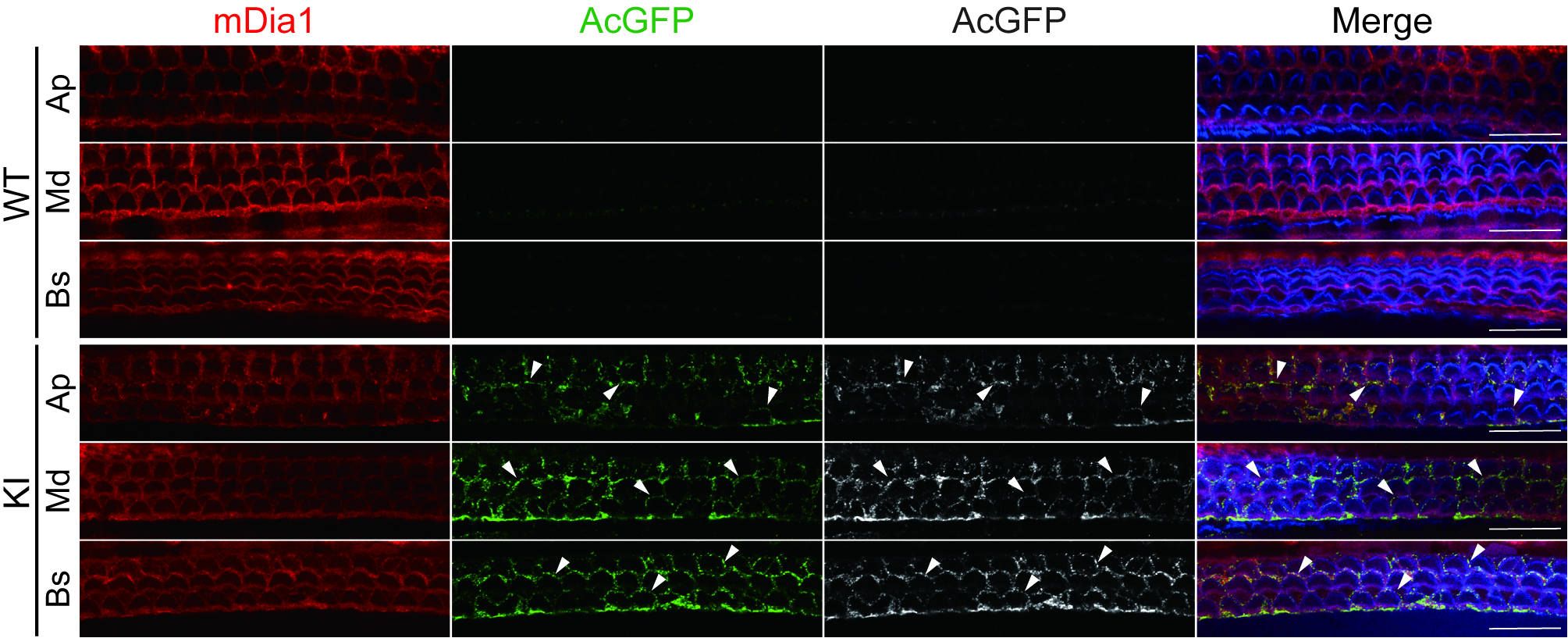

Supplement: Supplementary file 5 — Supplementary Figure S4 [file 41419_2020_2743_MOESM5_ESM.tif]
